# The impact of task-oriented training on hand functionality in children with cerebral palsy under 18 years: a systematic review and meta-analysis

**DOI:** 10.3389/fneur.2026.1775810

**Published:** 2026-03-05

**Authors:** Yuxin Xiao, Jun Zhang, Yongfu Liu, Xiaoyan Chen, Ruifeng Wu, Shenglong Le, Wei Fan, Le Zhao, Feng Gao

**Affiliations:** 1 Taihe Hospital, Hubei University of Medicine, Shiyan, Hubei, China; 2 Department of Assistive Devices, Taihe Hospital Affiliated to Hubei University of Medicine, Shiyan, Hubei, China; 3 Department of Acupuncture, Taihe Hospital Affiliated to Hubei University of Medicine, Shiyan, Hubei, China; 4 Department of Rehabilitation, Taihe Hospital Affiliated to Hubei University of Medicine, Shiyan, Hubei, China; 5 Department of Physical Therapy, Taihe Hospital Affiliated to Hubei University of Medicine, Shiyan, Hubei, China; 6 Department of Physical Education, Shanghai Jiao Tong University, Shanghai, China

**Keywords:** cerebral palsy, children, hand function, meta-analysis, task-oriented training

## Abstract

**Objective:**

Task-oriented training (TOT) is the predominant rehabilitative training approach grounded in motor control theory. The effectiveness of treating hand dysfunction in children with cerebral palsy has not been established. This research conducts a systematic analysis of the rehabilitation treatment effects of TOT on hand dysfunction in children with cerebral palsy.

**Methods:**

Comprehensive searches were performed in databases including China National Knowledge Infrastructure, Wan Fang Database, Chinese Science and Technology Periodical Databases, SinoMed, PubMed, Web of Science, Embase, and Cochrane Library for randomized controlled trials of TOT utilized in the rehabilitation of hand dysfunction in pediatric patients with cerebral palsy. The control group underwent standard rehabilitation treatment, but the experimental group received TOT alongside the control group treatment. The search time concluded in August 2025, coinciding with the establishment of each database. Two researchers independently performed literature screening and data extraction, while the quality of the literature was assessed using the Cochrane risk of bias assessment tool. A meta-analysis was conducted with Revman 5.4 software.

**Results:**

Sixteen publications were included, encompassing 1,037 children with cerebral palsy. The meta-analysis indicated that the enhancement of hand function in the experimental group surpassed that of the control group [SMD = 1.44, 95% CI (1.12, 1.76), *p* < 0.01]. Additionally, the recovery of grip strength and cognitive ability was superior in the experimental group [SMD = 0.52, 95% CI (0.17, 0.87), *p* < 0.05; MD = 0.93, 95% CI (0.60, 1.27), *p* < 0.01]. Furthermore, the WeeFIM Scale score for the experimental group exceeded that of the control group [MD = 6.47, 95% CI (5.22, 7.72), *p* < 0.01].

**Conclusion:**

TOT can boost the manual dexterity of children with cerebral palsy, and improve their grip strength and cognitive abilities. It is also more efficacious in enhancing the functional autonomy of youngsters. It is important to highlight that certain markers of the overall sample size, such as grip strength and IQ, were featured in only three publications, resulting in inadequate statistical power. Future research necessitates further large-sample, high-quality randomized controlled trials to validate the aforementioned conclusions.

## 1 Introduction

Cerebral palsy (CP) is a collection of conditions associated with limb disabilities, primarily defined by motor dysfunction, atypical posture and behavior, and restrictions in activities. The onset is frequently linked to non-progressive cerebral injury occurring during the fetal or infantile stage (1, 2). CP can be classified into several types, among which spastic CP accounts for approximately 80% of all CP cases (3). Research indicates that around 60% of children with CP experience hand function impairments and movement coordination challenges, specifically characterized by diminished grasping ability, inadequate bimanual coordination, and suboptimal fine motor control, significantly limiting their engagement in daily activities (4–6). Hand function is a crucial means of individual contact with the environment, and the extent of its recovery is closely correlated with the self-care capacity, learning aptitude, and social integration skills of children with CP. Consequently, enhancing the hand function of children with CP to increase their capacity for daily tasks has emerged as a primary focus of contemporary research.

The prevalent techniques employed in clinical practice to enhance hand function in children with CP encompass acupuncture, physical therapy, and occupational therapy. While traditional intervention methods can facilitate the recovery of motor function to some degree, they possess limitations including a narrow range of treatment modalities, insufficient engagement and interactivity, and a lack of personalization, which adversely impact children’s motivation to participate and the sustainability of long-term therapeutic outcomes (7–9). As rehabilitation medicine advances, an increasing number of innovative rehabilitation training models have been integrated into clinical practice. TOT, a strategy focused on functional scenarios, has increasingly garnered attention. TOT establishes precise and attainable task objectives aligned with the children’s actual capabilities, incorporates training into authentic contexts, and facilitates the acquisition and transfer of motor skills through repetitive, targeted, and significant task training, thereby enhancing the adaptation and reorganization of the neuromuscular system and improving the upper limb motor proficiency of the children (10–12).

Nonetheless, the quantity of randomized controlled trials (RCTs) regarding the implementation of TOT in the rehabilitation of hand function for children with CP is presently constrained, and the sample sizes are typically little, with a lack of systematic evidence synthesis (13–15). This study systematically extracted pertinent RCTs from both Chinese and English databases via a meta-analytic approach, amalgamated existing clinical research data and increased the sample size through quantitative synthesis. It aims to systematically evaluate the efficacy of TOT in the hand function rehabilitation of children with CP, and further explore the impact of factors such as intervention duration and assessment instruments on effect sizes through subgroup analysis to clarify the applicability of TOT under different clinical conditions, thereby providing evidence-based support for the clinical implementation of TOT in the rehabilitation of children with CP.

## 2 Data methods

This systematic review and meta-analysis adhered to the protocols established by the Cochrane Handbook for Systematic Reviews of Interventions. This research protocol has been filed on the PROSPERO platform of the National Institute for Health Research in the United Kingdom for prospective systematic reviews, registration number CRD420251161543.

### 2.1 Inclusion criteria

The criteria for inclusion in this systematic review were as follows: (1) Literature type: RCTs of TOT for the treatment of children with CP were included in this study; (2) Research subjects: Individuals diagnosed with CP via clinical assessment, aged under 18 years, with no restriction on gender, race, ethnicity or geographic region (16); (3) Intervention measures: The control group received conventional rehabilitation treatment; the experimental group received TOT in addition to the treatment of the control group; (4) Study language: Chinese and English; (5) Outcome indicators: Including hand function, grasping strength, intelligence, and functional independence. Among them, hand function was evaluated by the Peabody Motor Development Scale (PDMS-2), the Box and Block Test (BBT), the Assisting Hand Assessment (AHA), and the Fine Motor Function Measure Scale (FMFM) methods.

### 2.2 Exclusion criteria

The excluded studies were the following: (1) Republished studies; (2) Research data cannot be quantitatively combined and specific data cannot be obtained by contacting the authors; (3) The research design was a non-randomized or cohort study; (4) Unable to obtain the full text or the literature only has the abstract; (5) Reviews, dissertations, case analyses, case reports, conference abstracts, etc.

### 2.3 Database and retrieval strategy

The databases China National Knowledge Infrastructure, Wan Fang Database, Chinese Science and Technology Periodical Databases, SinoMed, PubMed, Embase, Web of Science, and Cochrane Library were carefully searched for RCTs published to August 2025 regarding the effect of TOT on hand function recovery in children with CP. A search approach employing a combination of subject headings and free-text terms was utilized, and the references of the selected literature were also examined. This search strategy and inclusion criteria was conducted in strict adherence to the PRISMA guidelines. The search criteria encompassed “CP, TOT, task-oriented, task-orientated therapy.” The comprehensive search approach for PubMed database is presented in Supplementary Table S1. Additionally, the reference lists of all included articles were manually screened to identify any potentially eligible studies not captured by the electronic search.

### 2.4 Data collection and extraction

Two researchers independently evaluated the retrieved literature based on the inclusion and exclusion criteria, extracted data, and verified the data. In the event of a disagreement between the two, a third researcher was consulted to decide on its inclusion. The retrieved literature data encompassed author, publication year, sample size, age, gender, intervention measures, intervention duration, and outcome indicators, among others.

### 2.5 Risk of bias assessment

Two researchers independently evaluated the included literature in accordance with the latest revised version of the risk of bias assessment tool (ROB) 2.0 recommended by Cochrane (17). The assessment covered the following 7 items: (1) generation of random sequences; (2) concealment of allocation; (3) blinding of subjects and interventionists; (4) blinding of outcome assessors; (5) completeness of outcome indicator data; (6) possibility of selective reporting of research results; (7) other sources of bias. The outcome indicators were classified as “low risk of bias,” “high risk of bias,” or “unclear.” The scores for each field were all “Low,” indicating low risk of bias; if at least one field was rated as “Some concerns,” and there were no “High risk” scores in any of the fields, it was considered medium risk of bias; if at least one field was rated as “High risk,” it was considered high risk of bias. After the assessment was completed, the two researchers cross-checked the evaluation results. In case of disagreement, the third researcher would make the final decision. Although inter-rater agreement (e.g., Cohen’s *κ*) was not statistically calculated, consensus was reached on all final judgments after independent evaluation to ensure methodological rigor and reliability. Furthermore, to ensure objectivity in the assessment process, information such as the authors, journals, and publication years of all included studies was concealed prior to evaluation.

### 2.6 Statistical analysis

Data analysis was performed utilizing RevMan 5.4 software. All outcome indicators were continuous variables. When same measurement tools were employed, the weighted mean difference (WMD) served as the effect size; conversely, when disparate measurement tools were utilized, the standardized mean difference (SMD) was applied as the effect size. The 95% confidence interval (CI) was employed for interval estimation. The variability among the studies was assessed using the *I*^2^ test and the *Q* test. When *p* > 0.1 and *I*^2^ < 50%, it signifies that the studies are homogeneous, warranting the employment of a fixed-effect model for pooling; conversely, when *p* ≤ 0.1 and *I*^2^ ≥ 50%, it denotes heterogeneity among the studies, necessitating the application of a random-effect model for pooling. When heterogeneity was substantial, sensitivity analysis or subgroup analysis was employed to ascertain its source. The statistical methods in this study have been evaluated by biostatistics experts at Taihe Hospital, affiliated to Hubei University of Medicine.

## 3 Results

### 3.1 Study screening

**Figure 1:**
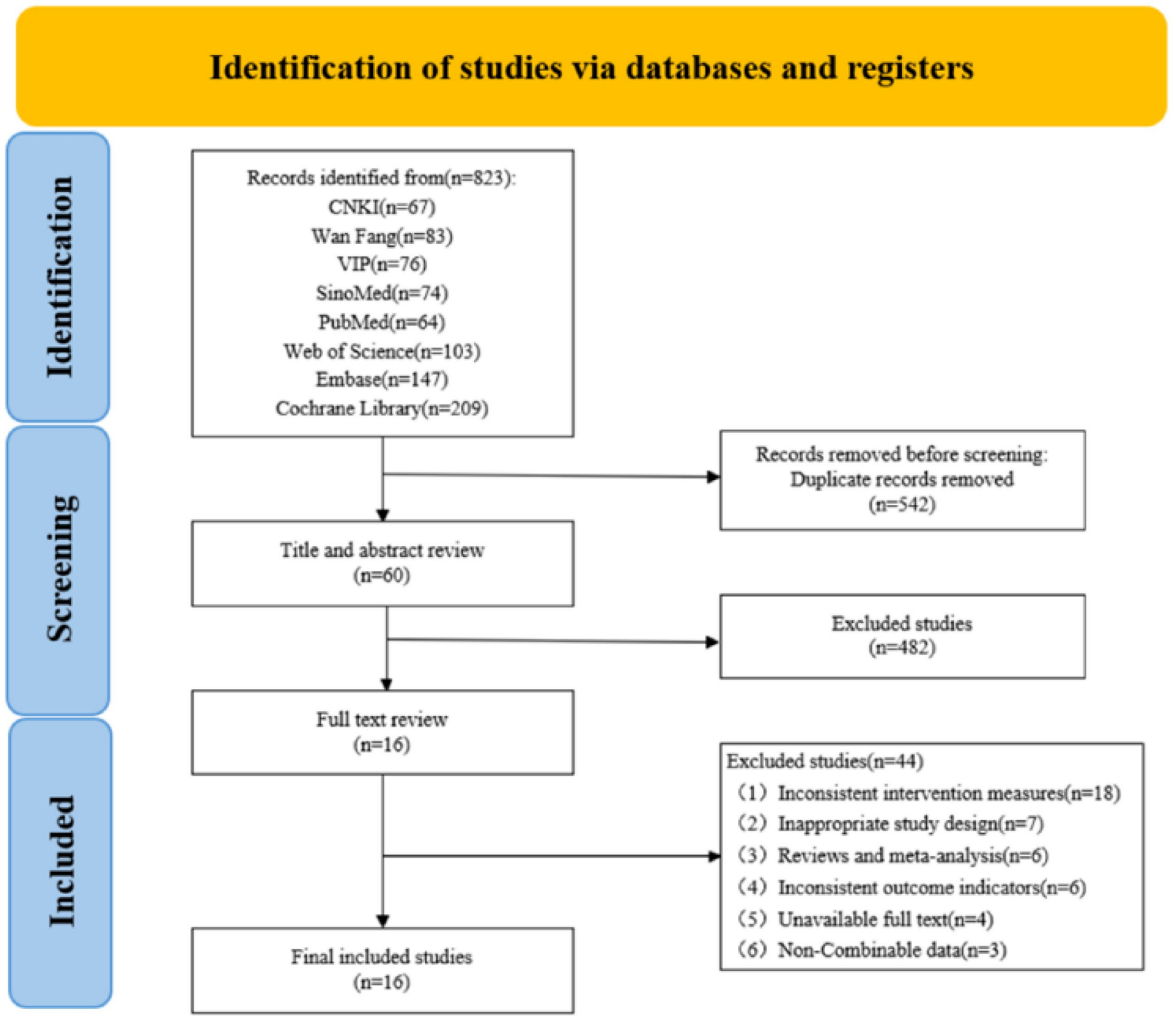
Flow chart of the search and selection process.

The preliminary search produced 823 pertinent documents. After the elimination of duplicates, 542 documents persisted. Following the review of titles and abstracts, 482 papers were excluded, resulting in a total of 60 documents remaining. Upon reviewing the complete texts, a total of 16 documents were ultimately included (18–33), among which 14 were in Chinese (18, 20–32) and 2 were in English (19, 33). The specific screening process of the documents is shown in Figure 1.

### 3.2 Characteristics of included studies

The research encompassed were published from 2015 to 2025, comprising a total of 1,037 children diagnosed with CP. The experimental group comprised 520 participants, whereas the control group included 517; there were 575 males and 462 females. All patients were under 18 years of age, and the intervention duration ranged from 1.5 to 6 months. In the 16 study outcome indicators, exist nine (18, 19, 21, 25–28, 31, 32) hand function status comparisons, three (19, 23, 33) grip strength value comparisons, three (20, 24, 30) intelligence value comparisons, and seven (22–25, 29, 30, 32) WeeFIM Scale comparisons. The basic characteristics of the included studies are shown in Table 1.

**Table 1:** Basic characteristics table of included literature.

Study	Sample T/C	Age T/C	Types of CP (SCP/HCP/other)	Sex (male/female) T/C	Intervention		Training frequency	Treatment duration (months)	Outcome indicator
					Trial	Control			
Fan et al. (18)	12/12	T: 50.83 ± 13.75 m	T: 12/0/0	T: 5/7	Task-oriented game music + conventional occupational therapy	Conventional occupational therapy	40 min/time, 5 times/wk	2	①
		C: 51.02 ± 13.5 m	C: 12/0/0	C: 6/6					
Moon et al. (19)	6/6	T: 58.1 ± 7.7 m	T: 6/0/0	T: 4/2	TOT + conventional occupational therapy	Conventional occupational therapy	20 min/time, 2 times/wk	2	②⑤
		C: 57.3 ± 3.4 m	C: 6/0/0	C: 2/4					
Chen et al. (20)	30/30	T: 4.6 ± 1.3 y	T: 30/0/0	T: 17/13	TOT + bobath therapy	Bobath therapy	40 min/time	3	⑦
		C: 4.5 ± 1.1 y	C: 30/0/0	C: 16/14					
Tang et al. (21)	14/13	T: 5.3 y	T: 14/0/0	T: 9/5	TOT + conventional occupational therapy	Conventional occupational therapy	20 min/time, 5 times/wk	3	①
		C: 4.8 y	C: 13/0/0	C: 6/7					
Liu (22)	55/55	T: 5.36 ± 0.53 y	T: 37/0/18	T: 27/28	TOT + guided health education	Guided health education	40 min/time, 5 times/wk	3	⑧
		C: 5.24 ± 0.52 y	C: 35/0/20	C: 29/26					
Zhao et al. (23)	44/43	T: 4.88 ± 0.52 y	T: 0/44/0	T: 26/18	TOT + conventional rehabilitation + mirror feedback therapy	Conventional rehabilitation treatment + mirror feedback therapy	30 min/time, 5 times/wk	3	⑤⑧
		C: 4.69 ± 0.49 y	C: 0/43/0	C: 25/18					
Lv et al. (24)	40/40	T: 4.29 ± 0.86 y	T: 40/0/0	T: 22/18	TOT + conventional rehabilitation training	Conventional rehabilitation training	5 times/wk	Front and back	⑥⑧
		C: 4.17 ± 0.92 y	C: 40/0/0	C: 21/19					
Li et al. (25)	20/20	T: 52 ± 8 w	T: 20/0/0	T: 11/9	TOT	Conventional rehabilitation treatment	30 min/time, 6 times/wk	3	①⑧
		C: 54 ± 10 w	C: 20/0/0	C: 12/8					
Wu et al. (26)	30/30	T: 5.84 ± 3.12 y	T: 30/0/0	T: 19/11	TOT + conventional rehabilitation training	Conventional rehabilitation training	—	3	①
		C: 5.93 ± 2.79 y	C: 30/0/0	C: 17/13					
Zhang et al. (27)	62/61	T: 4.70 ± 0.62 y	T: 61/0/0	T: 37/24	TOT + conventional rehabilitation treatment + biofeedback technology	Conventional rehabilitation treatment + biofeedback technology	40 min/time, 6 times/wk	4	②
		C: 4.50 ± 0.59 y	C: 62/0/0	C: 39/23					
Mao et al. (28)	30/30	T: 42.77 ± 10.90 m	T: 30/0/0	T: 17/13	TOT + conventional treatment methods + hydrotherapeutics	Conventional treatment methods + hydrotherapeutics	40 min/time, 5 times/wk	3	③
		C: 41.57 ± 11.26 m	C: 30/0/0	C: 12/18					
Fan et al. (29)	31/31	T: 7.75 ± 1.23 y	T: 31/0/0	T: 19/12	Family-centered TOT + family rehabilitation training	Family rehabilitation training	60 min/time, 5 times/wk	6	⑧
		C: 8.00 ± 1.38 y	C: 31/0/0	C: 21/10					
Li et al. (30)	43/43	T: 4.20 ± 1.50 y	T: 43/0/0	T: 25/18	TOT	Conventional rehabilitation training	—	3	⑧
		C: 4.30 ± 1.30 y	C: 43/0/0	C: 23/20					
Cao et al. (31)	50/50	T: 3.52 ± 0.67 y	T: 50/0/0	T: 28/22	TOT + conventional rehabilitation treatment	Conventional rehabilitation treatment	20~30 min/time, 5 times/wk	3	①
		C: 3.49 ± 0.64 y	C: 50/0/0	C: 27/23					
Chen et al. (32)	38/38	T: 6.11 ± 1.60 y	T: 38/0/0	T: 19/19	TOT + sling exercise training	Sling exercise training	40 min/time, 5 times/wk	3	①⑧
		C: 6.10 ± 2.42 y	C: 38/0/0	C: 21/17					
Daliri et al. (33)	15/15	T: 7.60 ± 1.18 y	T: 15/0/0	T: 9/6	Virtual reality TOT + conventional occupational therapy	Conventional occupational therapy	—	1.5	⑤
		C: 7.20 ± 1.20 y	C: 15/0/0	C: 4/11					

① Hand function-PDMS-2; ② Hand function-BBT; ③ Hand function-AHA; ④ Hand function-FMFM; ⑤ Grip strength; ⑥ Intelligence-Wechsler intelligence scale; ⑦ Intelligence- China-Binet intelligence test manual; ⑧ Functional independence-WeeFIM. T, trial group; C, control group; SCP, spastic cerebral palsy; HCP, hemiplegic cerebral palsy; PDMS-2, the Peabody Motor Development Scale; BBT, the Box and Block Test; AHA, the Assisting Hand Assessment; FMFM, Fine Motor Function Measure Scale; WeeFIM, the functional independence measure for children.

### 3.3 Quality of evidence

**Figure 2:**
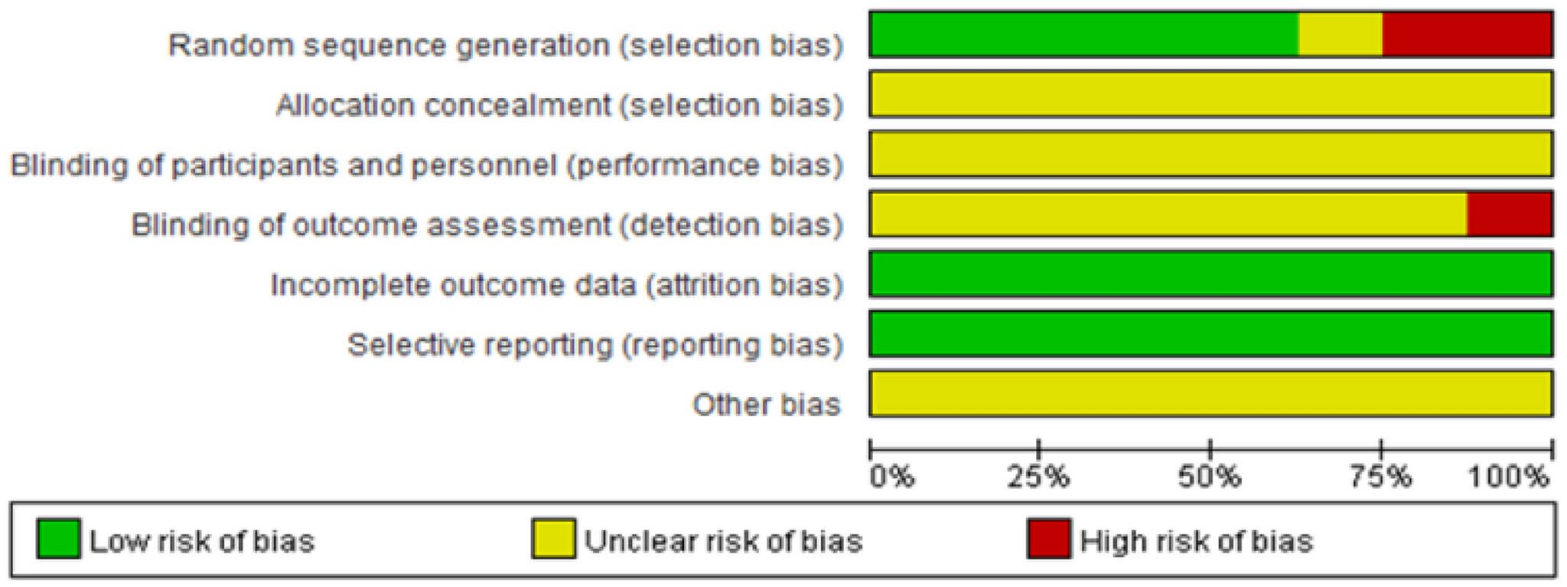
Risk of bias graph.

**Figure 3:**
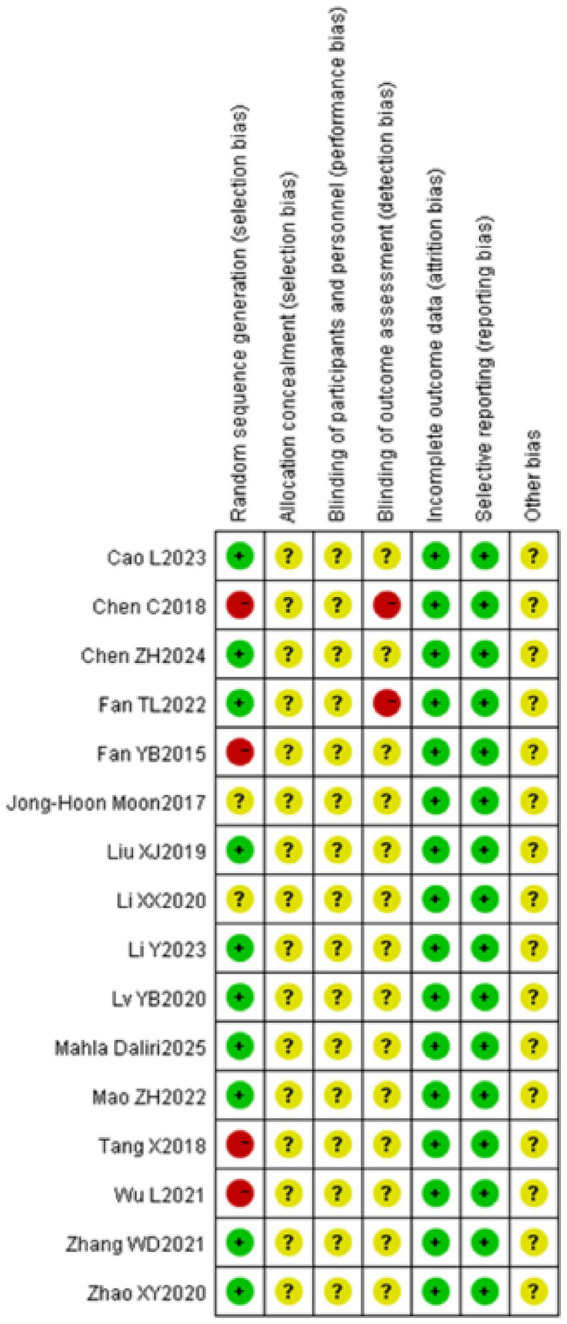
Risk of bias summary.

Two researchers employed the Cochrane risk of bias assessment technique to examine the quality of the seven dimensions of the included literature. Ten publications among them elucidated the methodology for creating the random sequence (22–24, 27–33), Six papers solely referenced randomization (18–21, 25, 26), whereas two articles employed blinding for the outcome assessors (20, 29). No literature reviewed exhibited absent outcome data or selective result reporting. The presence of additional causes of bias in the included literature was ambiguous. The methodological quality assessment of the included literature is shown in Figures 2, 3.

### 3.4 Meta-analysis results

#### 3.4.1 Hand function

**Figure 4:**
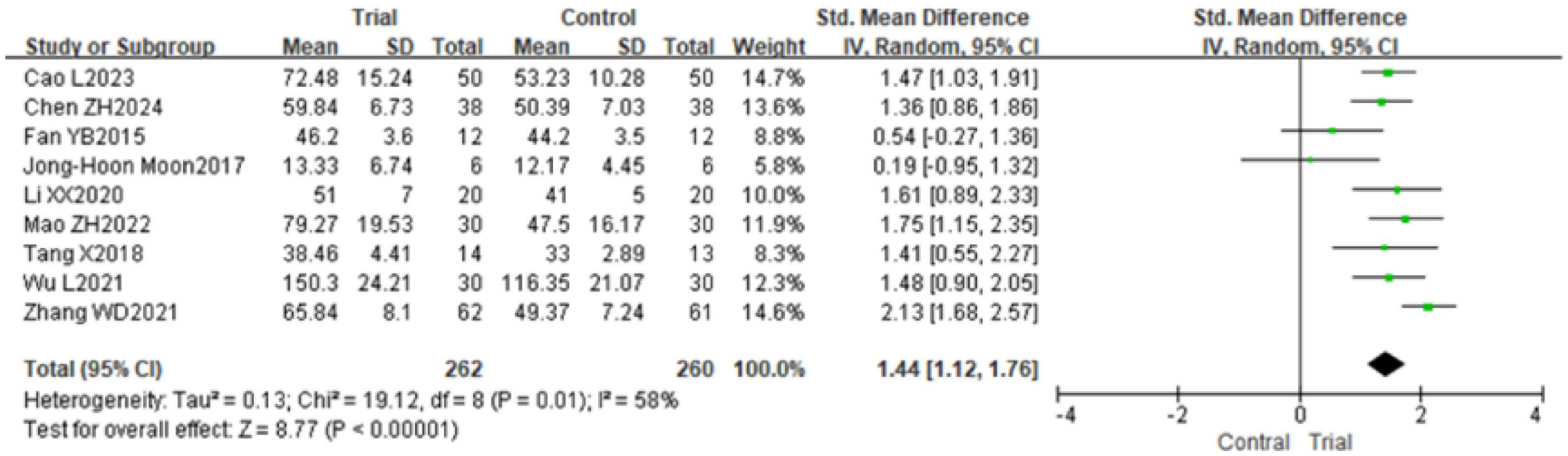
The forest plot of the effect of TOT on hand function. TOT, task-oriented training.

Nine studies included instruments such as PDMS-2, FMFM, AHA, and BBT to assess the hand function of children with CP from various perspectives (18, 19, 21, 25–28, 31, 32), pertaining to 522 children. This study assessed hand function utilizing various measurement instruments, including PDMS-2, FMFM, AHA, and BBT; hence, SMD was chosen as the effect size for the meta-analysis. The findings revealed considerable variability across the trials (*p* = 0.01, *I*^2^ = 58%), necessitating the application of a random effects model for analysis. The meta-analysis indicated that, relative to the control group, the hand function of children with CP in the experimental group exhibited a more pronounced improvement, with a statistically significant difference [SMD = 1.44, 95% CI (1.12, 1.76), *p* < 0.01]. Refer to Figure 4. Sensitivity analysis, conducted by sequentially excluding individual studies, revealed no significant alterations, hence demonstrating the robustness of the meta-analysis findings.

#### 3.4.2 Grip strength

**Figure 5:**
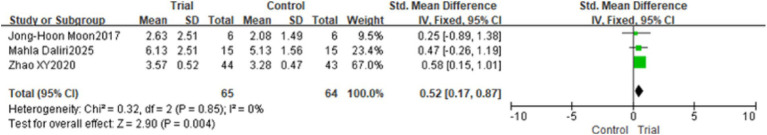
The forest plot of the effect of TOT on grip strength. TOT, task-oriented training.

Three studies assessed the grip strength of children with CP (19, 23, 33), pertaining to 129 children. The inclusion of this factor in only three studies resulted in an uneven weight distribution. Consequently, the SMD was employed to equilibrate the weights among the investigations. The meta-analysis results indicated no heterogeneity across the trials (*p* = 0.85, *I*^2^ = 0%), and a fixed-effect model was employed for the study. The meta-analysis revealed a statistically significant disparity in grip strength between the experimental group and the control group of children with CP [SMD = 0.52, 95% CI (0.17, 0.87), *p* < 0.05]. Refer to Figure 5.

#### 3.4.3 Intelligence

**Figure 6:**
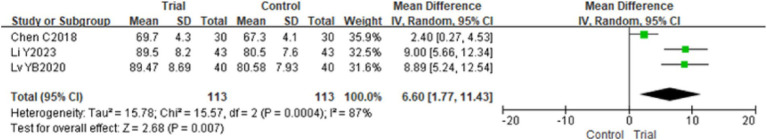
The forest plot of the effect of TOT on intelligence. TOT, task-oriented training.

Three studies assessed the IQ of children with CP (20, 24, 30), pertaining to 146 children. This article employed the Wechsler Intelligence Scale and the Chinese-Binet Intelligence Test Manual as measurement scales to evaluate children’s intelligence, so SMD was chosen as the effect size for the meta-analysis. The findings demonstrated minimal heterogeneity among the studies (*p* < 0.01, *I*^2^ = 32%), and a fixed-effect model was employed for the analysis. The meta-analysis indicated a statistically significant difference in IQ improvement between the experimental group of children with CP and the control group [MD = 0.93, 95% CI (0.60, 1.27), *p* < 0.01]. Refer to Figure 6.

#### 3.4.4 WeeFIM

**Figure 7:**
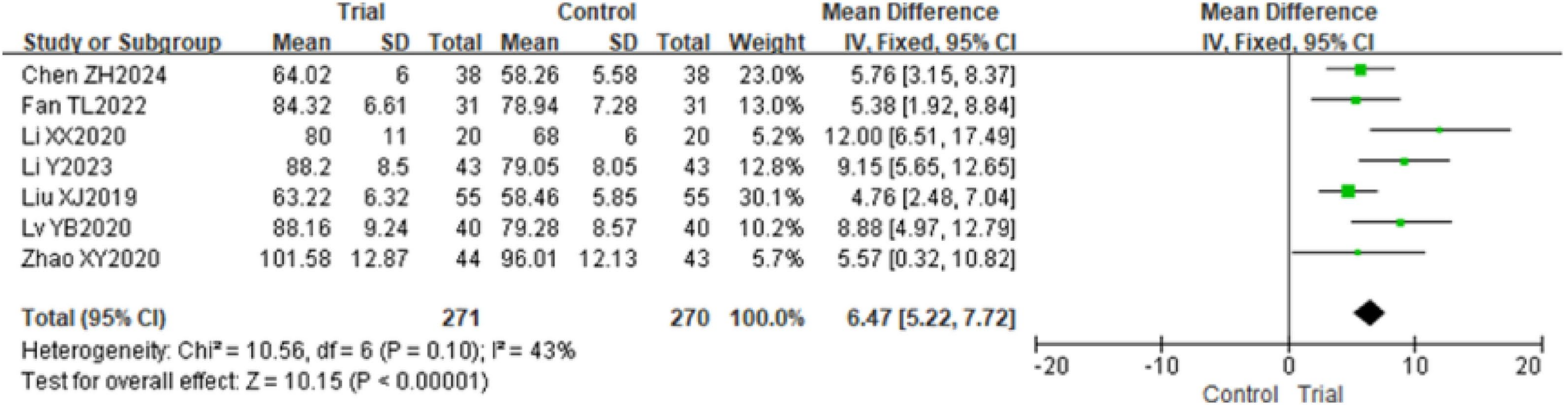
The forest plot of the effect of TOT on WeeFIM. TOT, task-oriented training, WeeFIM, Functional Independence Measure for Children.

Seven studies utilized the WeeFIM Scale to evaluate the independence of children with CP (22–25, 29, 30, 32), pertaining to 541 children. The findings indicated minimal heterogeneity among the studies (*p* = 0.10, *I*^2^ = 43%), and a fixed-effect model was employed for the analysis. The meta-analysis revealed that the WeeFIM scores of children with CP in the experimental group were significantly higher, with a mean difference of 6.47 [95% CI (5.22, 7.72), *p* < 0.01]. Refer to Figure 7.

### 3.5 Subgroup analysis

**Figure 8:**
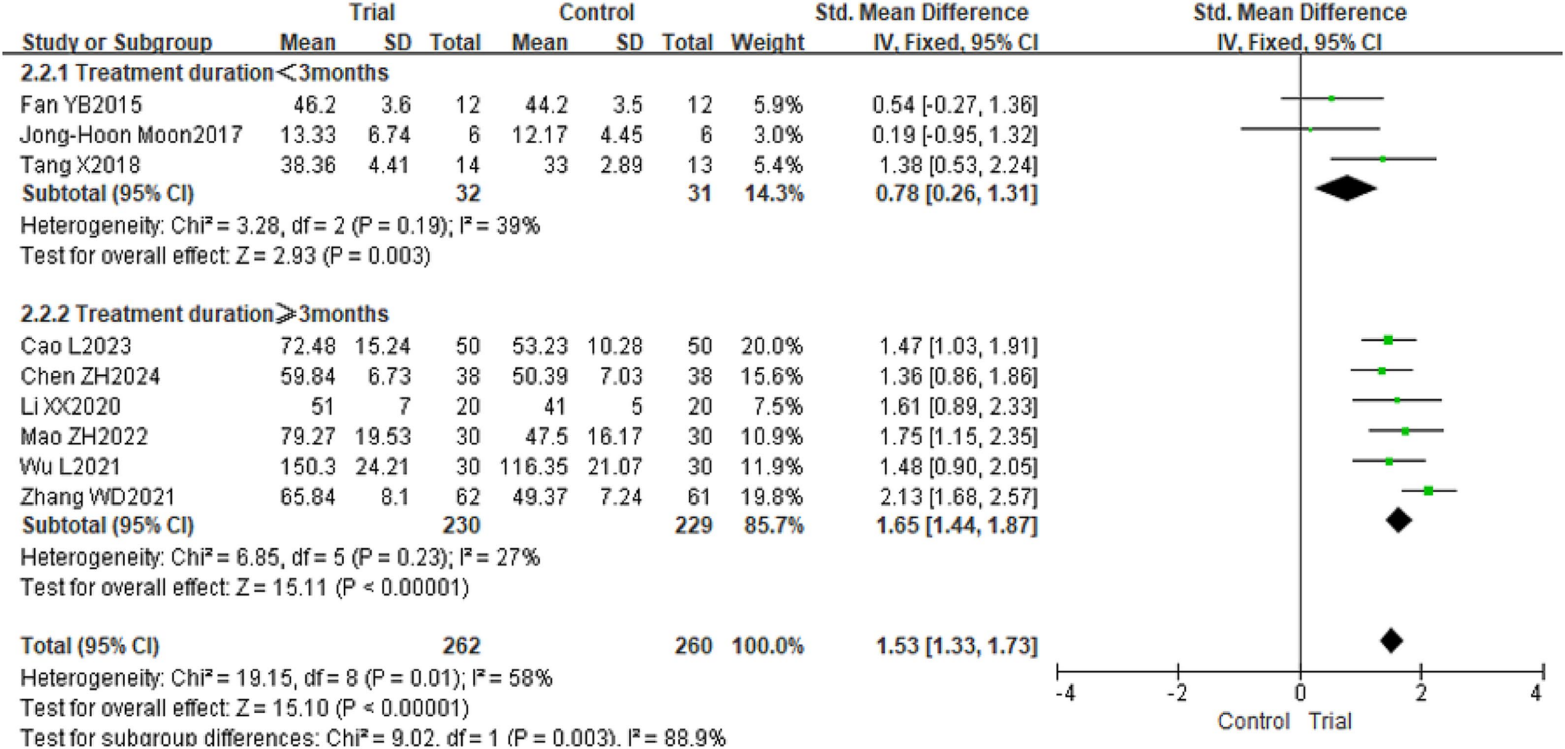
The forest plot of the subgroup analysis on intervention duration for TOT in improving hand function in children with CP. TOT, task-oriented training.

**Figure 9:**
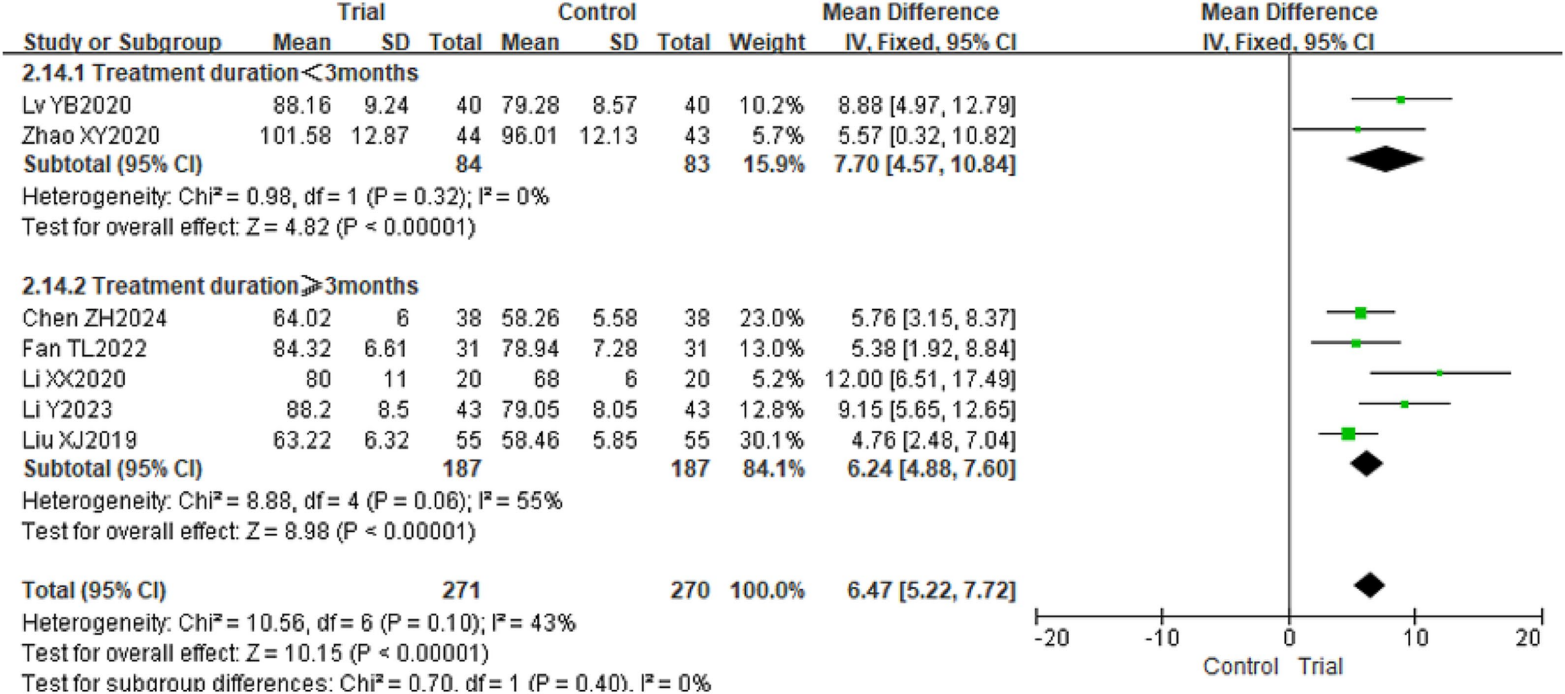
The forest plot of the subgroup analysis on intervention duration for TOT in improving functional independence in children with CP. TOT, task-oriented training.

Subgroup analyses were performed on hand function and WeeFIM scores according to the intervention period to augment the persuasiveness of the evidence in the literature with a substantial number of entries. The experimental group exhibited superior hand function improvement compared to the control group during both intervention periods: less than 3 months and 3 months or more. Notably, the enhancement in hand function for the experimental group was significantly greater in the 3 months or more category [Intervention period <3 months: SMD = 0.80, 95% CI (0.28, 1.33), *p* < 0.01; Intervention period ≥3 months: SMD = 1.65, 95% CI (1.44, 1.87), *p* < 0.01]. Refer to Figure 8; WeeFIM score: The functional independence recovery of the experimental group surpassed that of the control group during both the intervention period of less than 3 months and the intervention period of 3 months or more [Intervention period <3 months: MD = 7.70, 95% CI (4.57, 10.84), *p* < 0.01; Intervention period ≥3 months: MD = 6.24, 95% CI (4.88, 7.60), *p* < 0.01]. Refer to Figure 9.

**Figure 10:**
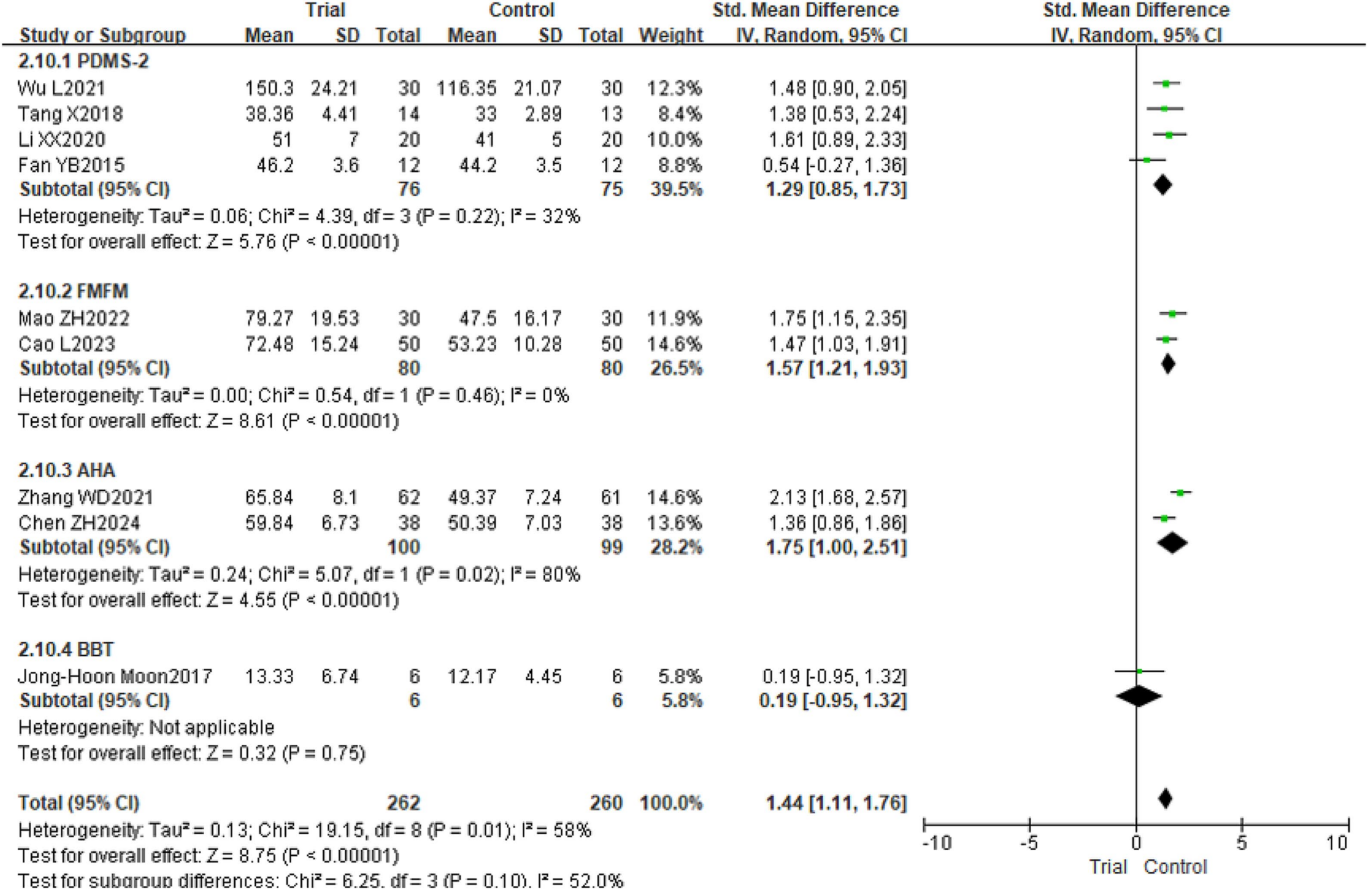
The forest plot of the subgroup analysis of hand function measurement instruments in children with CP. TOT, task-oriented training.

In addition, subgroup analysis based on different assessment instruments revealed that the experimental group scores were consistently higher than those of the control group when hand function was evaluated using PDMS-2, FMFM, or AHA. [PDMS-2: SMD = 1.29, 95% CI (0.85, 1.73), *p* < 0.01; FMFM: SMD = 1.57, 95% CI (1.21, 1.93), *p* < 0.01; AHA: SMD = 1.75, 95% CI (1.00, 2.51), *p* < 0.01]. Refer to Figure 10.

### 3.6 Publication bias

When a meta-analysis of outcome measures includes fewer than 10 studies, the use of funnel plots for publication bias analysis is not recommended. Therefore, only a qualitative assessment of publication bias was conducted. The included RCTs in this study had relatively small sample sizes, which may lead to a higher risk of publication bias.

## 4 Discussion

The advancement of biomedical technology has led to the implementation of numerous novel rehabilitation techniques aimed at enhancing hand functionality in children with CP. In addition to traditional approaches like exercise therapy, occupational therapy, and acupuncture, there are also balancing function training, core stability training, motor relearning, motor control theory, and TOT (34–36). TOT is a therapeutic approach designed to rectify the functional impairments of typical movements in children with CP. In comparison to alternative methods, TOT prioritizes the development of children’s problem-solving skills and their active engagement (19). This study carefully gathered RCTs examining the effects of TOT on hand functional impairments in children with CP, increased the sample size, and this meta-analysis encompassed 16 papers, comprising a total of 1,037 children with CP. The findings indicated that, in contrast to conventional rehabilitation techniques, TOT might markedly boost hand function, grip strength, cognitive abilities, and functional independence in children with CP.

TOT, as a task-oriented and functionally contextualized rehabilitation intervention method, can be categorized into two types based on its implementation form and task characteristics: functional task training grounded in real-life contexts and simulated task training situated in virtual or gamified contexts. Functional task training grounded in real-life contexts prioritizes the execution of significant tasks within authentic scenarios (e.g., handling tableware, writing), whereas simulated task training, utilizing virtual or gamified environments, replicates task scenarios through virtual reality or interactive games to augment the enjoyment and repetitiveness of the training. The mechanism of action of TOT primarily depends on motor control and motor learning theories, focusing on facilitating the adaptive rearrangement of the neuromuscular system and optimizing the corticospinal route through repetitive, goal-directed task practice (37, 38). It is important to recognize that real-life context-based TOT is more effective for improving children’s practical application skills in everyday activities, but virtual or gamified TOT offers distinct benefits in engaging children’s interest and delivering immediate feedback. Despite the differences in the two training methodologies, both emphasize task-oriented approaches and collaboratively enhance hand function.

The functionality of the hand is essential for daily tasks and is intimately linked to an individual’s quality of life. This study thoroughly examined the effect of TOT on the manual dexterity of children with CP. The findings indicated that the hand function enhancement in children within the TOT group surpassed that of the control group, implying that TOT can markedly enhance fine motor skills, hand coordination, and hand dexterity in children with CP. This aligns with the findings of Zhang’s (39) research. The proposed process may pertain to the enhancement of central nervous system flexibility and the reconstruction of brain function through TOT. TOT is founded on motor control theory and, through repetitive, TOT, enhances sensory-motor integration and the remodeling of the corticospinal pathway, thereby improving motor output and coordination in children, ultimately aiming to enhance upper limb motor function and action coordination (19, 40–42). In addition to hand dysfunction, children with CP often present with impaired limb motor function, balance control deficits, and intellectual abnormalities. These symptoms significantly hinder their normal growth and development processes and limit their levels of activity and participation in daily life (6, 43–45). As a task-guided comprehensive rehabilitation training technique, TOT serves as an effective means of reducing disability rates in children with CP. It employs a guided approach to deliver targeted, step-by-step task training based on the specific functional deficits exhibited by each child. It demonstrates clear clinical potential and practical significance in improving fine motor skills, coordination, and daily functional use of the hand in children with CP. In clinical practice, TOT can be integrated into individualized comprehensive rehabilitation programs, tailored to the specific functional characteristics and rehabilitation goals of each child. The adequacy of a specific duration of TOT for optimal functional recovery in children with CP warrants additional investigation. This study performed a subgroup analysis based on the duration of the intervention. When the intervention duration was under 3 months, a statistically significant difference was observed between the TOT group and the control group (*p* = 0.003); when the intervention duration was 3 months or longer, a significant statistical difference was noted (*p* < 0.01), demonstrating that the hand function improvement in the TOT group surpassed that of the control group. Consequently, it was concluded that TOT exerts a long-term effect on enhancing hand function in children with CP. The aforementioned results suggest that suitably prolonging the intervention duration of TOT can enhance therapeutic outcomes for children.

This study demonstrates that TOT can significantly improve grip strength in children with CP. The mechanism may arise from targeted training of grip strength control in the task, involving varying intensities and durations of grasping activities, which enhances the recruitment of motor units and muscle coordination patterns. Nonetheless, this indicator was incorporated into the study with relative infrequency, and one of the investigations exhibited an unusually high sample size, leading to the selection of SMD for the amalgamation of effect sizes to equilibrate weights. The results may exhibit certain biases. Additional research is required to validate the specific impact of TOT on enhancing grip strength in children with CP.

This study’s findings indicate that TOT positively influences the cognitive development of children with CP, aligning with Ji’s (46) research outcomes. This may result from TOT’s implementation of controlled training via active movements, which encourages central nerve cells to relocate to the lesion location, thereby continuously refining and optimizing the neural network and boosting the cognitive abilities of the youngsters. Nonetheless, the instruments employed to assess this variable differ (Wechsler Intelligence Scale/Chinese-Binet Intelligence Test Manual), hence SMD should be chosen as the impact size for the aggregated analysis. Two research employed the Wechsler Intelligence Scale for assessment, while just one study utilized the Chinese-Binet Intelligence Test Manual for evaluation. The variability in measurement instruments and the scarcity of research may undermine the reliability of the findings. Moreover, additional research with bigger sample sizes and standardized assessment instruments is necessary to elucidate the effect of TOT on the cognitive abilities of children with CP.

This study demonstrates that TOT positively influences the functional independence score (WeeFIM) of children with CP. This aligns with the findings of Li et al.’s (41) research. The mechanism suggests that TOT is fundamentally training derived from real-life activities, facilitating children’s effective transfer of acquired skills to everyday situations and improving practical abilities such as self-care and mobility (19). This study performed a subgroup analysis according to the timing of the intervention. The WeeDIM Scale scores of the TOT group with an intervention duration of less than 3 months and those with an intervention duration of 3 months or more exceeded those of the control group. This further demonstrates that TOT exhibits a markedly superior therapeutic impact compared to standard rehabilitation in enhancing the daily living skills of children, underscoring its extensive applicability in clinical practice.

This meta-analysis possesses specific limitations: (1) Only publicly available Chinese and English literature was retrieved, excluding grey literature; (2) The brain-damaged children in the included studies exhibited variability in disease duration, types of brain damage, severity, and intervention timing, resulting in a degree of heterogeneity; (3) Several included studies had small sample sizes, and only three studies were combined in the results, potentially leading to publication bias and compromising the quality of the meta-analysis; (4) The included studies exclusively involved minors and did not address adult patients with brain damage, which may introduce result bias; (5) The included literature generally failed to mention the implementation of blinding for subjects and interventionists, indicating low methodological quality; (6) Due to differences in rehabilitation infrastructure, health insurance policies, family participation models, and sociocultural support, the direct external validity of the findings to clinical settings in other countries and regions may be limited; (7) The combined nature of interventions in some studies makes it difficult to precisely isolate the independent effect of TOT, which may affect the evaluation of its independent efficacy.

## 5 Conclusion

This study concluded that TOT can boost the fine motor abilities, gripping ability, hand coordination, and hand flexibility of children with CP, augment their grasping power, and improve their cognitive function and functional independence. Nevertheless, the ideal intervention dosage, including frequency, intensity, and cycle, must still be ascertained on an individual basis. For children with spastic hemiplegia, TOT emphasizes fine motor training of the affected hand; for those with double or quadriplegia, greater emphasis should be placed on hand coordination and trunk stability. It is important to highlight that several markers of the overall sample size in this study (such as grip strength and intellect) were present in only three papers, resulting in inadequate statistical power. Consequently, the extrapolation of the findings should be approached with caution. Future research necessitates further large-sample, high-quality RCTs to validate the aforementioned result. Simultaneously, additional stratified analyses of the impact of TOT on the efficacy across various forms of CP, age demographics, and levels of functional impairment should be undertaken to develop more tailored rehabilitation strategies.

## Data Availability

The original contributions presented in the study are included in the article/Supplementary material, further inquiries can be directed to the corresponding authors.
