# Supplementary figures and images for: The impact of task-oriented training on hand functionality in children with cerebral palsy under 18 years: a systematic review and meta-analysis
Source: Front Neurol. 2026 Mar 5;17:1775810. doi: 10.3389/fneur.2026.1775810 (PMC12999552; doi:10.3389/fneur.2026.1775810)

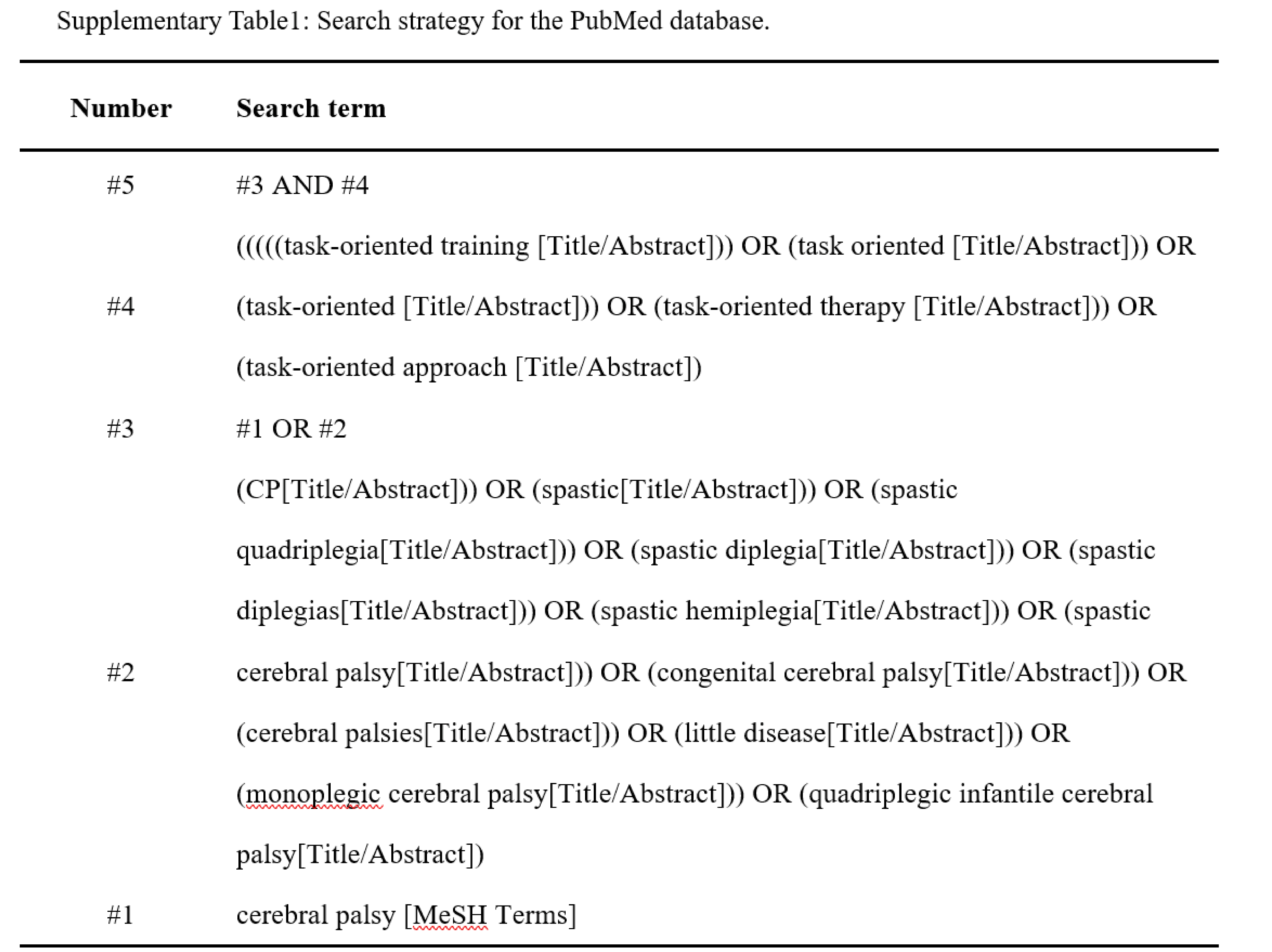

Supplement: Supplementary file 1 [file Image_1.tif]
